# An independent Taiwanese lineage of powdery mildew on the endemic host species *Koelreuteria henryi*

**DOI:** 10.1186/s40529-024-00431-1

**Published:** 2024-07-19

**Authors:** Yu-Wei Yeh, Roland Kirschner

**Affiliations:** 1 School of Forestry and Resource Conservation, National Taiwan University, Roosevelt Rd. Sec. 4 No. 1, Taipei City, Taiwan; 2 Mycology Research Group, Faculty of Biological Sciences, Goethe University Frankfurt Am Main, Biologicum, Max-Von-Laue-Straße 13, Frankfurt Am Main, Germany

**Keywords:** *Bulbouncinula*, Powdery mildew, Erysiphaceae, *Koelreuteria*, Co-speciation

## Abstract

**Background:**

Powdery mildews (Erysiphaceae, Ascomycota) are common plant disease agents and also cause stress for forest and fruit trees worldwide as well as in Taiwan. The powdery mildew *Erysiphe bulbouncinula* on *Koelreuteria* host trees was considered an endemic species in China. While in China the host was *K. paniculata* and only the teleomorph stage found, the anamorph and the teleomorph were both recorded for the host in Taiwan, *K. henryi*. We aimed to clarify the relationship of the powdery mildews recorded under *E. bulbouncinula* with an apparently disjunct distribution.

**Results:**

Specimens of powdery mildew on *K. henryi* from Taiwan were characterized based on the anamorph morphology and DNA sequences. They revealed a new record of *Sawadaea koelreuteriae* for this host species and Taiwan and a new species of *Erysiphe*, *E. formosana*, sister to *E. bulbouncinula* from China.

**Conclusions:**

In *Erysiphe* on *Koelreuteria* hosts, speciation of plant parasitic fungi seems to be correlated with disjunct host and geographic distribution possibly shaped by extinction of potential host species which are known only as fossils. Two of the three extant East Asian species of *Koelreuteria* are now known as hosts of specific *Erysiphe* species. We may predict a further not yet discovered *Erysiphe* species on the third East Asian species, *K. bipinnata*, in South and Southwest China. In the speciation in *Sawadaea*, the extinction events in *Koelreuteria* can be excluded from being involved.

## Background

Among the four extant species of *Koelreuteria* (Sapindaceae), *K. henryi* Dümmer is the single and endemic species in Taiwan (Chen 1993; Wang et al. 2013). In the past, this species was sometimes considered a subspecies of *K. elegans* A.C. Sm., *K. elegans* subsp. *formosana* (Hayata) F.G. Mey., while *K. elegans* A.C. Sm. subsp. *elegans* is distributed in Fiji (Wang et al. 2013). Due to morphological differences and geographical distances, the two taxa in Taiwan and Fiji are now treated as separate species (Chen 1993; Wang et al. 2013). *Koelreuteria henryi*, commonly known as Taiwanese golden rain tree, is the single species of *Koelreuteria* used as an ornamental landscape tree in Taiwan (Hsueh and Yang 2008) and was also introduced to the United States in 1915 and Australia around 2000 (Wang et al. 2013). *K. henryi* has been widely planted as a street tree or ornamental tree due to its beautiful flowers and adaptive abilities (Gilman et al. 2019). However, *K. henryi* has been recognized as a naturalized or invasive plant in many parts of the world, particularly in Australia, the USA and Japan (Meyer 1976).

Powdery mildews (Erysiphaceae) as economically important and worldwide distributed plant pathogenic fungi have the potential to serve as model for the spread of pathogens during climate change in the past and present (Glawe 2008). The morphological features of Erysiphaceae including anamorphic and teleomorphic ones are used as important evidence for species identification (Braun and Cook 2012). Molecular characterization of species of powdery mildews initiated by the work of S. Takamatsu, Japan, was not only a major breakthrough in new genus concepts (Braun and Takamatsu 2000), but also in the identification of powdery mildews found only in the anamorph stage which is dominant in tropical/subtropical areas and has few diagnostic morphological characteristics compared to the teleomorph (Braun and Cook 2012). Since then, sequences of the internal transcribed spacer (ITS) of the ribosomal RNA genes have become the principal DNA barcode for species identification and revealing new cryptic species (Bradshaw et al. 2023; Meeboon et al. 2016; Meeboon and Takamatsu 2016, 2017a, b, 2020; Takamatsu et al. 2015a, b).

*Koelreuteria* species are hosts of two species of powdery mildews, *Erysiphe bulbouncinula* U. Braun & S. Takam [*Bulbouncinula bulbosa* (F. L. Tai & C. T. Wei) R. Y. Zheng & G. Q. Chen] and *Sawadaea koelreuteriae* (I. Miyake) H.D. Shin & M.J. Park (Liu et al. 2020; Shin and Park 2011). While *S. koelreuteriae* is known from *K. paniculata* in China and Korea (Liu et al. 2020; Shin and Park 2011), *Erysiphe bulbouncinula* is a powdery mildew species recorded from two *Koelreuteria* species in China and Taiwan (Kuo 1993; Liu et al. 2020). In Taiwan, *E*. *bulbouncinula* on *K. henryi* (as “*K. elegans*”) was first recorded by Hsieh (1986) as *Uncinula clintonii* Peck and described in detail as anamorph and teleomorph under *Bulbouncinula bulbosa* by Kuo (1993). The taxonomic history of *E*. *bulbouncinula* was outlined by Liu et al. (2020). Braun and Cook (2012) provided the morphology of the teleomorph of *E. bulbouncinula* from China without considering the anamorph description and records from Taiwan by Hsieh (1986) and Kuo (1993). When we found an anamorphic powdery mildew on *K. henryi* in Taiwan and obtained an ITS sequence fitting to *Erysiphe*, we did not consider this confirmation of the generic classification in *Erysiphe* worth of publication. After Liu et al. (2020), however, confirmed the generic accommodation of *E. bulbouncinula* by phylogenetic analysis of newly collected specimens from China, we found that our sequence from Taiwan was quite different from those from China and hypothesized that the powdery mildew on the endemic Taiwanese *Koelreuteria* species may represent a lineage independent from that in China.

## Methods

### Sample collection and morphology

In this study, samples of Erysiphaceae were collected from different sites in northern Taiwan between winter 2017 and spring 2024 and processed immediately or kept in a refrigerator (ca. 8 °C) before processing. For light microscopical observation, fresh samples were removed from the plant surface with transparent tape, mounted in 5–10% KOH or water with a cover glass and observed under 1000× magnification. The sizes of conidiophores and conidia were measured and presented as mean value ± standard deviation of 20 or 30 measurements with extreme values in brackets. Drawing of the fungus was made by hand with scaled paper. Dried specimens mounted in KOH were used only for classifying the specimens on the generic level. The specimens were deposited in the National Museum of Natural Science, Taichung, Taiwan (TNM) after being dried by an electrical dryer.

### Molecular identification

The genomic DNA of the fungus was extracted from freshly collected conidia and mycelium; the PCR products were amplified, sequenced, and edited as in Wei and Kirschner (2017). The sequences of the internal transcribed spacer (ITS) region of the ribosomal RNA gene (including ITS 1, 5.8S rDNA, and ITS 2, and partial fragments of the flanking 18S and 28S rDNA) and of a part of the nuclear ribosomal large subunit RNA gene (LSU) were used for megaBLAST searches at GenBank and deposited in GenBank. The selected ITS sequences for an alignment were based on BLAST searches and Liu et al. (2020). The sequences were aligned with MEGA X with the default options with MUSCLE (Kumar et al. 2018) and minor manual adjustment of the distribution of gaps. The alignment was deposited in zenodo (10.5281/zenodo.11069494). The phylogenetic analyses were based on the Maximum Likelihood method with the Kimura-2 parameter model (gamma-distributed) as the best model and 1000 bootstrap replicates as in Yeh et al. (2023).

## Results

### DNA sequences comparison

BLAST searches with the ITS sequences revealed that two specimens belonged to *Sawadaea* and two other ones to *Erysiphe*. In the *Erysiphe* specimens, the ITS sequences of our two specimens R. Kirschner 4611 and 5942 were highly identical to each other, only one base pair was different. In the BLAST comparisons with sequences of similar lengths in GenBank, the ITS sequences from our materials had 96–97% identity (differing by 20–24 bps, including 7–10 gaps) with eight sequences of *E*. *bulbouncinula* (Liu et al. 2020), whereas other species had an identity of 89% or lower. The LSU gene sequence of our specimen R. Kirschner 5942 had an identity of 99% with eight sequences of *E*. *bulbouncinula* from Liu et al. (2020) with 4 different positions. The next most similar sequences were labelled as *E. mori* (I. Miyake) U. Braun & S. Takam. with an identity of 98%, and the other sequences were from Erysiphaceae with a lower identity.

In the *Sawadaea* specimens, our ITS sequences differed by 0–1 bp from all 16 sequences of *S. koelreuteriae* from China and South Korea in GenBank (99–100% identity), while other species had an identity of 96% or lower. Although comparatively small conidia (18 × 11 µm) were found in the specimens, no micro-conidiophores and micro-conidia were detected. By the lack of distinct dimorphism of conidia and conidiophores the specimens morphologically resembled *Podosphaera xanthii* (Castagne) U. Braun & Shishkoff, which has not been recorded from *Koelreuteria* hosts.

### Phylogenetic analysis

**Fig. 1:**
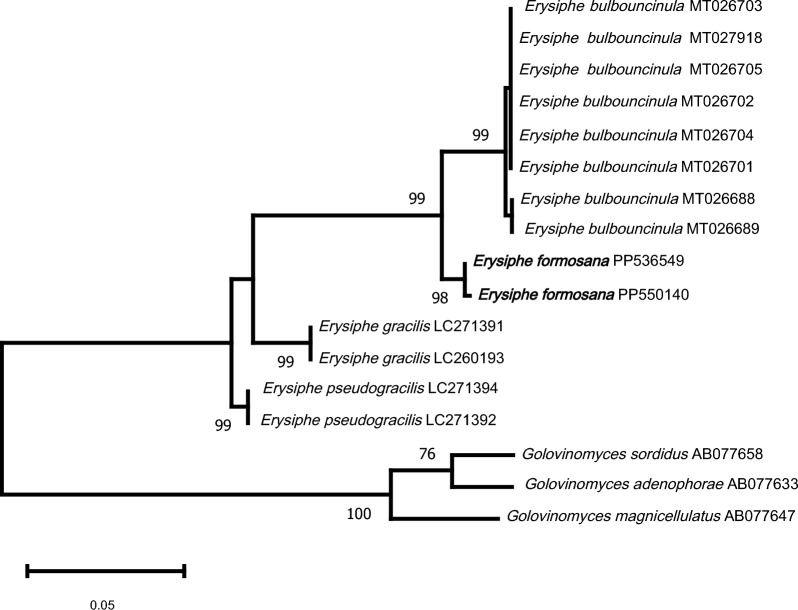
Unrooted maximum likelihood analysis of ITS sequences of *Erysiphe bulbouncinula*, *E. formosana*, and related Erysiphaceae species, with *Golovinomyces* spp. as outgroup and 1000 bootstrap replications

As shown in Fig. 1, the eight ITS sequences of *E. bulbouncinula* (MT026701–05, MT026688–89 & MT027918) from Liu et al. (2020) formed a clade with strong support, but were separated into two poorly supported subclades. Two sequences of our material from Taiwan (R. Kirschner 4611 & R. Kirschner 5942) formed a clade with strong support sister to the clade comprising *E. bulbouncinula* from China.

### Taxonomy

**Fig. 2:**
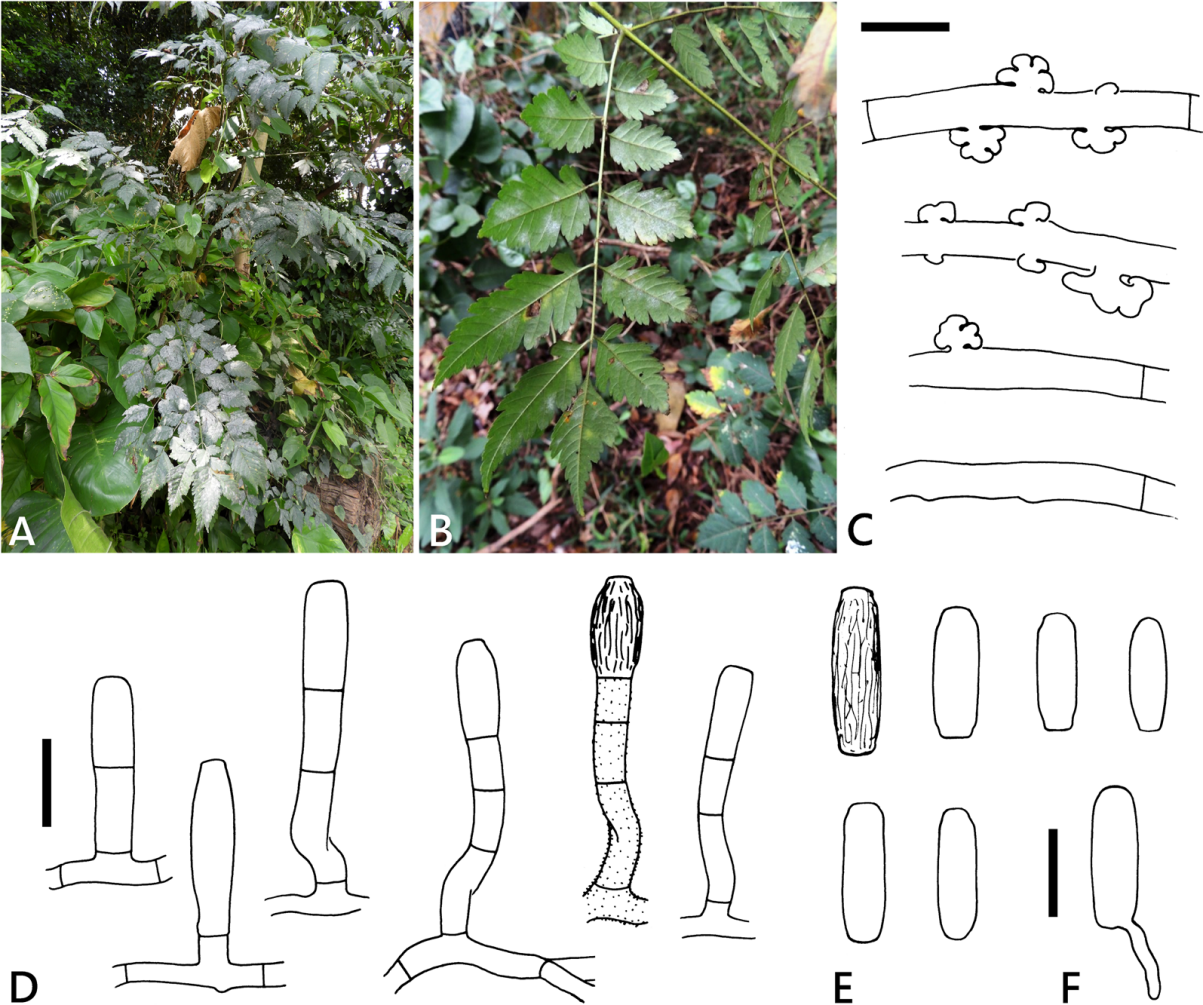
*Erysiphe bulbouncinula* on *Koelreuteria henryi* (R. Kirschner 4583, except A = R. Kirschner 4710). **A** Powdery mildew symptoms on abaxial side of leaves; **B** Powdery mildew symptoms on adaxial side of leaf; **C** Hyphae with appressoria; **D** Conidiophores with a straight or curved foot cell; **E** Conidia; **F** Conidium with germination hypha (surface ornament not shown in all conidiophores and conidia). Scale bars: C = 10 μm; all others = 20 μm

***Erysiphe formosana*** R. Kirschner & Yu-Wei Yeh, sp. nov. (Fig. 2).

Index Fungorum: IF 901254.

**Typification** TAIWAN: Hsinchu County, Hukou Old Street, ca. 24.8765 N, 121.0635 E, ca. 125 m, on seedlings of *Koelreuteria henryi* Dümmer, 17. Feb. 2018, R. Kirschner 4611 (TNM, **holotype!**), ITS GenBank PP536549.

**Etymology** Referring to the endemic distribution in Taiwan (“Formosa”) on the endemic tree *Koelreuteria henryi*, which also bears “formosana” in the subspecies name under its synonym *Koelreuteria elegans* subsp. *formosana*.

**Diagnosis** Differs from *E. bulbouncinula* by the dominance of the anamorph and by the host plant species.

Colonies amphigenous. Hyphae hyaline, verruculose, 3−6 µm wide. Hyphal appressoria nipple-shaped to lobed, solitary or in opposite pairs. Conidiophores verruculose to smooth, (30−)35−66(−75) × (6−)7−9(−10) µm (n = 20). Foot cell straight to distinctly curved, basal septum at the same level as upper surface of the conidiophore mother cell or raised above up to 8 µm, (12−)17−28(−30) × (5−)6−8(−9) µm (n = 20), followed by 0−2 further shorter cells. Conidia solitary, cylindrical, with fine longitudinal striation on the surface, 22−38(−46) × 9−11(−12) µm (n = 30), germinating at one or both ends with a short hypha without or with lobed appressorium.

Ascomata not seen. For teleomorphic characteristics see Kuo (1993).

**Additional specimens examined (paratypes)** On leaves of seedlings and mature trees of *Koelreuteria henryi*, TAIWAN: Hsinchu County, Hukou Old Street, ca. 24.8765 N, 121.0635 E, ca. 125 m, 31 Dec. 2017, R. Kirschner 4583 (TNM); Taipei City, Beitou District, Daoxiang Rd., ca. 25.144146 N, 121.488100 E, ca. 60 m, 14 Apr. 2019, R. Kirschner 4710 (TNM); Taipei City, Daan District, Fuzhoushan Park, ca. 25.016292 N, 121.553091 E, ca. 70 m, 5 Jan. 2024, R. Kirschner & Y.-H. Yeh 5942 (TNM), ITS GenBank PP550140, LSU GenBank PP548294; Taipei City, Daan District, National Taiwan University, at life science building, ca. 25.015802, 121.539190, ca. 10 m, 15 May 2024, R. Kirschner 6027 (TNM).

**Fig. 3:**
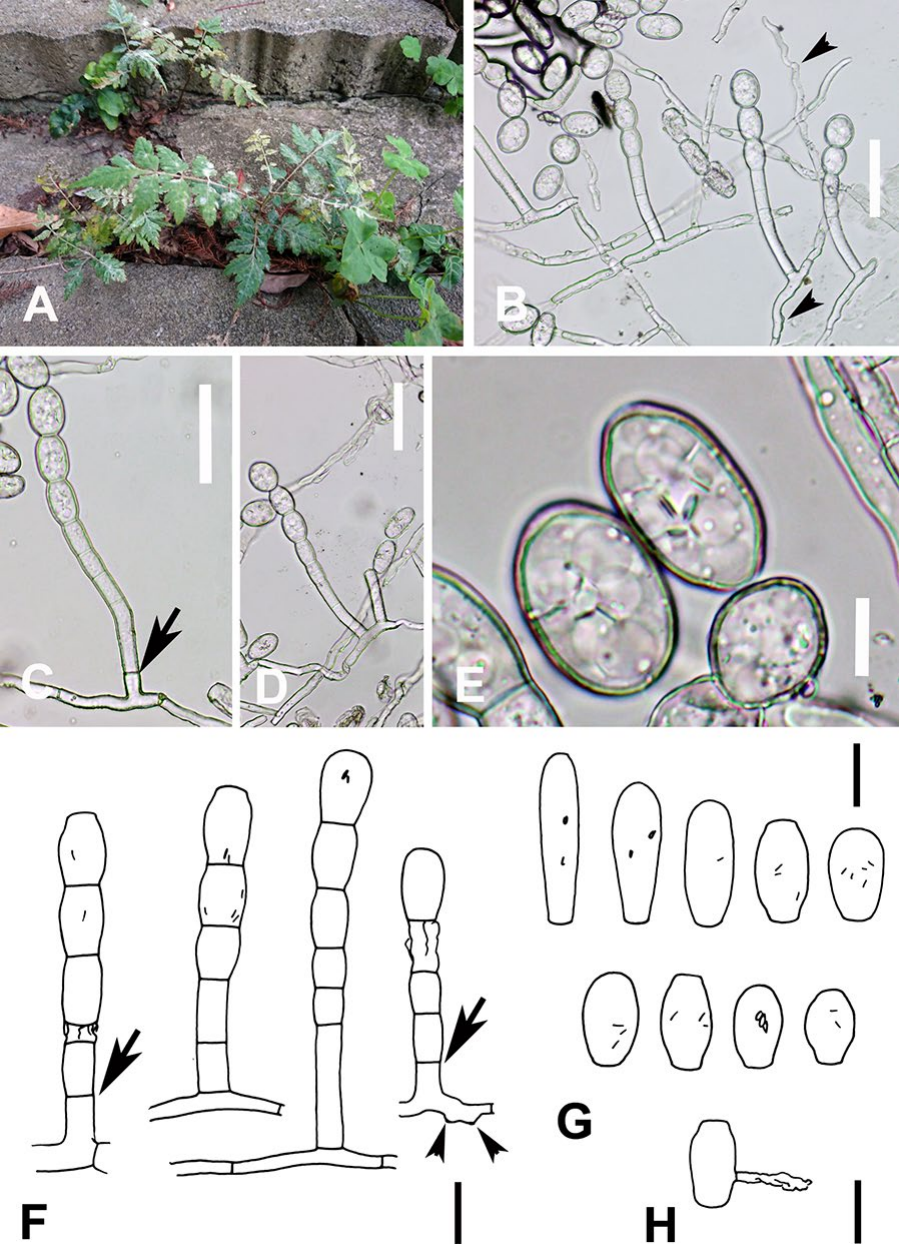
*Sawadaea koelreuteriae* on *Koelreuteria henryi* **A**–**E** R. Kirschner 5979, **F**–**H** R. Kirschner 5979-B). **A** Powdery mildew symptoms on seedlings; **B** Conidiophores arising from hyphae with nipple-shaped appressoria (arrowheads); **C** Conidiophore with basal septum (arrow) at some distance from the hyphal mother cell; **D** Two conidiophores arising from the same hyphal mother cell; **E** Conidia with fibrosin bodies; **F** Conidiophores. Basal septum raised above hypha indicated by arrow, nipple-shaped appressoria by arrowheads; **G** Conidia with fibrosin bodies; **H** Germinating conidium. Scale bars B, C, D = 50 μm; E = 10 μm; F, G, H = 20 μm

***Sawadaea koelreuteriae*** (I. Miyake) H.D. Shin & M.J. Park, J. Microbiol. 49(5): 864 (2011) (Fig. 3).

(Description based on fresh material of R. Kirschner 5979-B.) Colonies on stems, petioles and amphigenous on leaf laminas. Hyphae hyaline, smooth to verruculose, 4−9 µm wide. Hyphal appressoria inconspicuous to nipple-shaped. Macro-conidiophores arising singly from conidiophore mother cell, rarely 2 from the same hyphal cell, smooth or verruculose at base. Foot cell straight, basal septum at the same level as upper surface of the conidiophore mother cell or raised above up to 20 µm, (15−)20−38(−40) × 9−10 µm (n = 20), followed by conidia at different stages of development. Macro-conidia catenescent, ovoidal, ellipsoidal, pyriform, cylindrical, or clavate, smooth on the surface, containing fibrosin bodies, (25−)26−42(−55) × (11−)15−19(−22) µm (n = 30), smallest conidia 18 × 11 µm. Conidium germination near the middle with a short hypha without discernable appressorium. Micro-conidiophores and micro-conidia not found.

**Specimens examined** On young leaves and shoot apex of seedlings of *Koelreuteria henryi*, TAIWAN: Taoyuan City, Pingzhen District, Xinshi Park, ca. 24.9512 N, 121.2176 E, ca. 135 m, 6 May 2018, R. Kirschner 4635 (TNM); Taipei City, Daan District, National Taiwan University, Department of Horticulture and Landscape Architecture, Keelong Rd. Sec. 4, no. 138, 25.014626 N, 121.539702 E, ca. 10 m, 14 Mar. 2024, R. Kirschner 5979 (TNM), ITS GenBank PP727207; ibid., 16 Apr. 2024, R. Kirschner 5979-B (TNM); Taipei City, Datong District, MRT Yuanshan Station, ca. 25.069638, 121.519810, ca. 10 m, 12. May 2024, R. Kirschner 6026 (TNM), ITS GenBank PP809226.

## Discussion

### Molecular identification

The identification of Erysiphaceae to species level is traditionally based on the combination of sexual morphology and host specificity. Therefore, we initially followed Kuo (1993) in identifying the *Erysiphe* specimens from *Koelreuteria henryi* as *E. bulbouncinula*. Since Liu et al. (2020) provided new data about *E. bulbouncinula* on *K. paniculata* from China, more data became available for comparison. While in the ITS sequences, there was 1 bp difference between the two samples of *E. formosana* and 2 or 3 different bps among the specimens of *E. bulbouncinula* (Liu et al. 2020), the ca. 20 bps difference between both species was significant. These differences were correlated with strongly supported clades. The sequences of the LSU gene which is considered a relatively conservative region were identical among all samples of *E. bulbouncinula* (Liu et al. 2020) but had 4 different positions compared to *E. formosana*.

### Morphology and phenology

We did not find micro-conidiophores and micro-conidia in our specimens of *Sawadaea koelreuteriae* in three fresh specimens from Taipei City collected within ca. two months or in a dried specimen from a more distant place (Taoyuan City, R. Kirschner 4635) that we had initially misidentified as *E. bulbouncinula*. Although in the dried material conidiophores were collapsed, up to four short cells following the foot cell of some conidiophores and the absence of lobed appressoria indicated that this specimen was rather *S. koelreuteriae*. The dimensions of the macro-conidia were similar to the conidia of *Podosphaera xanthii*. As observed by Shin and Park (2011) in *S. koelreuteriae* from Korea and in our specimens, the basal transversal septum of the conidiophores is often shifted up to 20 µm above the hyphal mother cell. In *P. xanthii,* the basal septum is usually formed at the same level as the upper surface of the hyphal mother cell (Meeboon et al. 2016) and only exceptionally raised above for up to 10 µm (Yeh et al. 2021). The lack of micro-conidiophores in some specimens of *Sawadaea* spp. was also mentioned by Bolay (2005) and Homma (1937).

According to the descriptions of the teleomorph of the *Erysiphe* specimens by Kuo (1993) and Liu et al. (2020), there are no significant morphological differences. Although there is little doubt that the fungus described by Kuo (1993) belongs to *E. formosana*, we only refer to the teleomorph description by reference to Kuo (1993). The teleomorph-anamorph connection should be further confirmed by DNA data from ascomata (Liu et al. 2020). The anamorph morphology of *E. formosana* was highly similar to Kuo’s description (1993), including the often conspicuously curved foot cell and striate conidium surface, and is complemented here by giving the size of the conidiophore foot cell. The lack of a basal septum of the foot cell as illustrated in Kuo (1993) was not confirmed, but the occurrence of the raised basal septum above the level of the hyphal surface appears to be an important characteristic. Liu et al. (2020) in spite of extensive collection could not find conidia and thus doubted whether a conidial stage exists at all in *E. bulbouncinula*. Our specimens on *K. henryi* were collected from December to May, those of Hsieh (1986) and Kuo (1993) in January and November, respectively. Kuo (1993) found the conidial and ascomatal stages in the same specimen apparently collected in the central mountains of Taiwan above 800 m (Nantou County, “Wanda”). The other specimens in Taiwan were collected at lower altitudes. The collection period in Taiwan overlapped with that of October to November in China (Liu et al. 2020). If a conidial stage exists in *E. bulbouncinula* in China but has not yet been found, we still suggest that it is not as dominant as in *E. formosana* in Taiwan, where in contrast to China the teleomorph is rarely found. According to our field observation, the disease seems to be more apparent on young trees, particularly seedlings, than on mature trees. Hsieh (1986) mentioned yellowing and premature leaf dehiscence due to strong infection.

### Example of speciation in powdery mildews

The taxonomy of Erysiphaceae is re-examined and re-arranged with the support and complement of phylogenetic data, especially in some genera/species that lacked molecular data (Bradshaw et al. 2023). As in *E. formosana*, anamorphic characteristics are often better correlated with molecular cladistics than teleomorph features (Braun and Takamatsu 2000; Kirschner et al. 2020). Geographic and ecological isolation seems to be the major driving force in speciation in powdery mildews (Takamatsu 2013; Troch et al. 2014). The discovery of *E. formosana* on an endemic *Koelreuteria* species in Taiwan confirms this view for a narrow case of an island-endemic host plant, where geographic isolation may have led to speciation in the host plant followed by an ecological isolation and co-speciation of its parasite. Similar cases may be *E. densa* Berk. in New Zealand, *E. carpinicola* (Hara) U. Braun & S. Takam. in Japan and the recently discovered *E. canariensis* M. Bradshaw, U. Braun & V. Kumm. on an endemic plant on the Canary Islands (Bradshaw et al. 2023). Less specific East Asian endemism seems also to be correlated with speciation in powdery mildews such as in *Sawadaea koelreuteriae* on two host species in China, Korea, and Taiwan. Since *K. paniculata* naturally also occurs in Japan (Jiang et al. 2019), discovery of *E. bulbouncinula* and *S. koelreuteriae* could also be expected there. *Sawadaea* is a small genus, with some species described from East Asia still known only by their teleomorph morphology (Braun and Cook 2012) so that phylogeographic conclusions would be premature.

## Conclusions

This example of co-speciation within powdery mildews contributes to the present international efforts to complement our knowledge of “the world’s most familiar (yet poorly known) plant pathogens” (Glawe 2008). *Koelreuteria* species in the past were more widely distributed, with several extinct fossil species known from the Cenozoic Europe, North America, and Asia (Jiang et al. 2019; Wang et al. 2013), which became extinct most likely through climate change (ice ages, lifting of the Tibetan plateau). These geologically relatively recent extinction events in the potential host species may have exerted some pressure on their pathogens like powdery mildews which were forced to modify their original host range by co-speciation or host-jump. Based on our hypothesis of speciation of powdery mildews on *Koelreuteria* species being strongly influenced by extinction of potential host species, we may predict a further not yet discovered *Erysiphe* species on the third East Asian *Koelreuteria* species, *K. bipinnata* Franchet, in South and Southwest China. *Sawadaea* species may follow another adaptive strategy, which was not influenced by the extinction of *Koelreuteria* hosts but which in terms of species diversity may appear less successful.

## Data Availability

Deposit of data and materials is given in the Methods Section.
